# An intranasal combination vaccine induces systemic and mucosal immunity against COVID-19 and influenza

**DOI:** 10.1038/s41541-024-00857-5

**Published:** 2024-03-21

**Authors:** Man Xing, Gaowei Hu, Xiang Wang, Yihan Wang, Furong He, Weiqian Dai, Xinyu Wang, Yixin Niu, Jiaojiao Liu, Hui Liu, Xiaoyan Zhang, Jianqing Xu, Qiliang Cai, Dongming Zhou

**Affiliations:** 1 Department of Pathogen Biology, School of Basic Medical Sciences, Tianjin Medical University, Tianjin, 300070 China; 2 Shanghai Public Health Clinical Center, Fudan University, Shanghai, 201508 China; 3 MOE&NHC&CAMS Key Laboratory of Medical Molecular, Frontiers Science Center of Pathogenic Microorganisms and Infection, School of Basic Medical Sciences, Shanghai Medical College, Fudan University, Shanghai, 200032 China; 4 MOE&NHC&CAMS Key Laboratory of Medical Molecular Virology, Shanghai Institute of Infections Disease and Biosecurity, Frontiers Science Center of Pathogenic Microorganisms and Infection, School of Basic Medical Sciences, Shanghai Medical College, Fudan University, Shanghai, 200032 China; 5 Chengdu Kanghua Biological Products Co., Ltd, Chengdu, China

**Keywords:** Viral infection, Viral infection

## Abstract

Despite prolonged surveillance and interventions, the severe acute respiratory syndrome coronavirus 2 (SARS-CoV-2) and influenza viruses continue to pose a severe global health burden. Thus, we developed a chimpanzee adenovirus-based combination vaccine, AdC68-HATRBD, with dual specificity against SARS-CoV-2 and influenza virus. When used as a standalone vaccine, intranasal immunization with AdC68-HATRBD induced comprehensive and potent immune responses consisting of immunoglobin (Ig) G, mucosal IgA, neutralizing antibodies, and memory T cells, which protected the mice from BA.5.2 and pandemic H1N1 infections. When used as a heterologous booster, AdC68-HATRBD markedly improved the protective immune response of the licensed SARS-CoV-2 or influenza vaccine. Therefore, whether administered intranasally as a standalone or booster vaccine, this combination vaccine is a valuable strategy to enhance the overall vaccine efficacy by inducing robust systemic and mucosal immune responses, thereby conferring dual lines of immunological defenses for these two viruses.

## Introduction

Respiratory viral infections are among the most common diseases in humans worldwide, causing a serious global health and economic burden. Severe acute respiratory syndrome coronavirus 2 (SARS-CoV-2) and influenza viruses are the two leading causes of respiratory viral infections with high morbidity and mortality. Coronavirus disease 2019 (COVID-19) is caused by SARS-CoV-2, a novel coronavirus isolated in late 2019. As of October 18, 2023, 771,407,825 cases of COVID-19 and 6,972,152 deaths had been confirmed worldwide by the World Health Organization (WHO) Coronavirus Dashboard, although these figures are dramatically underestimated. Acute respiratory illnesses caused by influenza viruses have occurred for centuries in the form of pandemics, epidemics, outbreaks, and sporadic cases^1^. Annual influenza epidemics result in ~ billion infections, 3 – 5 million cases of severe illness, and 29,000 – 650,000 deaths^2^.

Vaccination is the primary strategy for combating infectious diseases. COVID-19 vaccines were available in 2021, and ~68.9% of the global population has received at least one dose of the vaccine as of September 2023, resulting in decreased rates of severe illness and mortality^3^. However, the COVID-19 pandemic has not been terminated by widespread vaccination; new variants, such as the currently circulating Omicron variant, continue to emerge. Waves of worldwide morbidity, hospitalization, and mortality have accompanied the emergence of new variants of concern (VOC) that have enhanced rates of transmission and/or ability to evade pre-existing SARS-COV-2 immunity^4^. Vaccination schedules have failed to keep pace with the pandemic, and booster doses are being used to improve protection against symptomatic and severe disease. Both SARS-CoV-2 and influenza virus thwart vaccination efforts due to waning vaccine- or infection-acquired immunity and their rapid and frequent mutation that evade pre-existing immunity. Presently, the influenza virus is the only pathogen for which annual vaccination is recommended. For the COVID-19 vaccine, the latest WHO recommendations is that high priority-use groups and sub-populations with special considerations should be revaccinated at 6–12 months after their previous dose^5^. Wiemken, T. L. et al. found that COVID-19 cases follow a similar seasonal pattern to other respiratory viruses, with transmission peaking during the winter despite continuing throughout the year, and supported employing annual preventative measures against SARS-CoV-2, such as seasonal booster vaccinations in a timeframe similar to that of influenza^6^. If it subsequently proves to be true, vaccination campaigns against both infections can be combined to reduce the total intervention cost; thus, a combination vaccine can be crucial. Moreover, the willingness to receive COVID-19 booster doses is significantly lower than that for primary vaccinations^7–10^. Combining influenza vaccines with COVID-19 booster doses might increase uptake of the latter since many populations are accustomed to receiving annual influenza vaccines^11,12^. Since the single, combination vaccine would offer protection against COVID-19 and influenza, it is clearly a better option for economically and effectively managing the pandemic.

SARS-CoV-2 and influenza viruses primarily infect the respiratory tract and spread through respiratory droplets and aerosols; therefore, the viral load within the oropharynx is an important determinant of disease transmission^13–15^. Currently, most of the approved COVID-19 and influenza vaccines are administered intramuscularly. These intramuscular (i.m.) vaccines elicit a potent systemic immune response but poor mucosal immunity localized in the respiratory tract, which may explain their suboptimal effectiveness in preventing infection^16,17^. Mucosal vaccination that induces robust local mucosal immunity in the respiratory tract may be critical in preventing viral infection, replication, and shedding and therefore, transmission^18^. Mucosal vaccines for COVID-19 and influenza are a logical approach to reduce viral infection and transmission.

Therefore, based on chimpanzee adenovirus serotype-68 (AdC68)^19^, we developed an intranasal (i.n.) combination vaccine, AdC68-HATRBD, with dual specificity against SARS-CoV-2 and influenza A virus, and evaluated the immunogenicity and protective potency of one or two doses in mice. We also investigated the potential of the combination vaccine as a heterologous booster following the licensed COVID-19 and influenza vaccine administration. Overall, we aimed to develop a convenient, affordable, and effective combination vaccine against COVID-19 and influenza.

## Results

### Design and construction of the combination vaccine AdC68-HATRBD

**Fig. 1:**
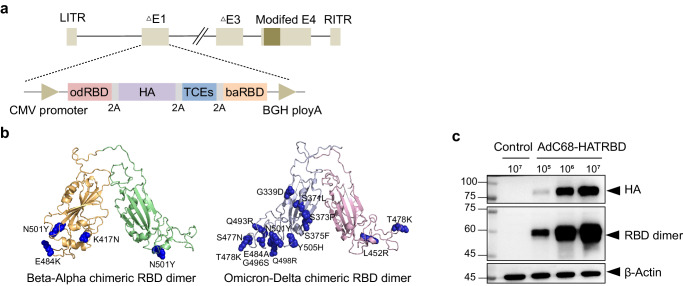
Design and construction of the combination vaccine AdC68-HATRBD. **a** Diagram of AdC68-HATRBD construction. odRBD, Omicron-Delta chimeric RBD dimer; baRBD, Beta-Alpha chimeric RBD dimer; TCEs, tandem conserved T-cell epitopes from ORF1,ORF3, and M proteins of SARS-CoV-2. **b** The sequence of baRBD or odRBD was subjected to AlphaFold2 server for tertiary structure prediction, and analyzed using PyMol. In baRBD dimer, Beta RBD subunit is shown in orange with Alpha RBD in green. In odRBD dimer, Omicron RBD subunit is shown in grey with Delta RBD in pink. **c** Western blot analysis of expression of RBD dimers and HA in AdC68-HATRBD transduced HEK 293 cell lysates under reductive conditions. AdC68-empty was included as negative control.

SARS-CoV-2 infects cells via the receptor-binding domain (RBD) of the spike protein, which binds to the cell receptor angiotensin-converting enzyme 2 (ACE2). Human influenza A viruses initiate infection by binding hemagglutinin (HA) to salivary acid receptors on the surface of respiratory epithelial cells. Previous studies demonstrated the role of RBD- and HA-based vaccines in inducing neutralizing antibodies (NAbs) that protect against viral infections^20,21^. Based on the chimpanzee adenoviral vector AdC68, we designed a combination vaccine, AdC68-HATRBD, carrying dual antigens of SARS-CoV-2 and influenza A virus. This vaccine contains two chimeric RBD dimers, numerous T cell epitopes (TCEs) of SARS-CoV-2, and full-length HA of A/California/07/2009 (pH1N1) (Fig. 1a).

A COVID-19 protein subunit vaccine ZF2001, based on the tandem homo-prototype RBD-dimer of the SARS-CoV-2 spike protein, confers 87.6% protection against severe to critical COVID-19^22^. The RBD dimers are much more immunogenic than RBD monomers, since RBD protomers are in a dimer stack on top of each other via the core subdomains, exposing the RBM, the primary site recognized by neutralizing antibody^23^. Given that SARS-CoV-2 has been constantly evolving, the induction of immunity against current VOCs alone is insufficient to protect against future VOCs. We hope to cover as many VOCs as possible with the two chimeric RBD-dimers strategy, thereby inducing broad-spectrum neutralizing antibodies to afford protection against current and future VOCs. Specifically, the C-terminal Beta RBD was linked to the N-terminal Alpha RBD, forming a Beta-Alpha chimeric dimer (baRBD), and the C-terminal Omicron BA.1 RBD was linked to the N-terminal Delta RBD, forming an Omicron-Delta chimeric dimer (odRBD). Figure 1b displays the predicted tertiary structures of chimeric RBD dimers. To broaden cellular immunity, immunogenic TCEs were selected from the structural and nonstructural proteins of ancestral SARS-CoV-2, which are conserved across VOCs and involved in T-cell activation against COVID-19^24–26^. Western blot analyses revealed dose-dependent effect of the vaccine on gene expression of chimeric RBD dimers and HA in vitro (Fig. 1c).

### The prime-boost regimen induces superior humoral immune responses

Intranasal vaccine delivery is an effective, non-invasive route of immunization. To investigate the distribution of vaccines administered intranasally, mice were immunized intranasally with 5 × 10^7^ infectious units (IFU) of AdC68-HATRBD, and lungs and nasal turbinates were collected to quantify vector genome load (Supplementary Fig. 1a). PBS-treated mice served as controls. Compared to controls, viral loads in the lungs of AdC68-HATRBD-treated mice were at baseline levels, whereas those in the nasal turbinates were as high as 2.4 × 10^8^ copy numbers/g (Supplementary Fig. 1b), indicating that the vaccine is dominantly distributed in the upper respiratory tract rather than in the lungs.

**Fig. 2:**
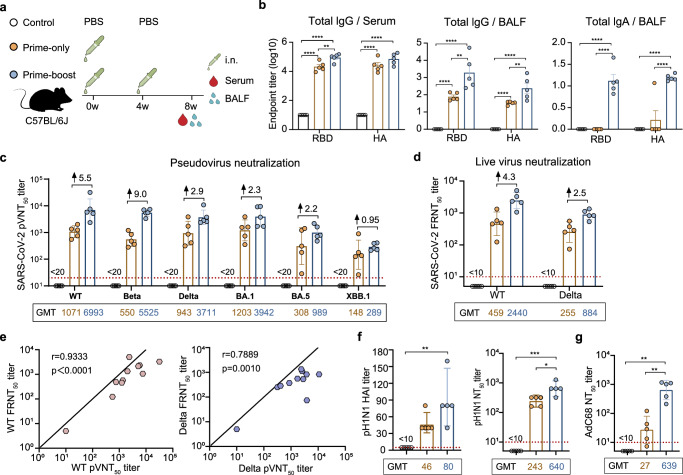
The prime-boost regimen induces superior humoral immune responses. **a** Scheme of experiments. Mice (*n* = 10 per group) were immunized intranasally with 5 × 10^7^ IFU of AdC68-HATRBD in prime-only or prime-boost regimen. The control group received a placebo (PBS). Serum and BALF were harvested at 8 week post-vaccination. **b** Binding antibody titers of the serum IgG (left), BAFL IgG (middle), and BAFL IgA (right) to RBD or HA, expressed in log10. **c** Serum pseudovirus neutralizing antibody against SARS-CoV-2 and VOCs. **d** Serum live virus neutralizing antibody against WT and Delta. **e** Spearman correlations of pVNT50 titer and FRNT50 titer against WT (left) and Delta (right). **f** Antibody titers against pH1N1 by the hemagglutination inhibition assay (left) and neutralization assay (right). **g** Serum neutralizing antibody against AdC68 vector. Each dot represents data from two mice. The red dashed line representing the lower limit of detection and geometric mean titer (GMT) values are displayed in (**c**), (**d**), (**f**), and (**g**). In (**c**) and (**d**), the numbers after the up arrow indicate the median fold increase in antibody titer induced by prime-boost regimen compared with prime-only regimen. Data are represented as mean ± SEM (**b**) or GMT ± SD (**c**, **d**, **f**, and **g**). Significance was assessed by one-way ANOVA with Tukey correction. *P ≤ 0.05, **P ≤ 0.01, ***P ≤ 0.001, ****P ≤ 0.0001.

We first evaluated the humoral immunogenicity of AdC68-HATRBD as an i.n. vaccine. Mice were immunized with 5 × 10^7^ IFU of AdC68-HATRBD using a prime-only or prime-boost regimen (Fig. 2a). Humoral immune responses to vaccination were determined by NAbs and binding antibodies (BAbs). Eight weeks post-vaccination (wpv), both prime-only and prime-boost regimens elicited RBD- and HA-specific immunoglobin (Ig) G antibodies in the serum and bronchoalveolar lavage fluid (BALF) (Fig. 2b). Compared to the prime-only regimen, the prime-boost regimen induced a substantial increase in the magnitude of IgG antibody responses. For mucosal IgA in the BALF, the prime-only regimen failed to generate anti-RBD and -HA IgA antibodies, while the prime-boost regimen significantly mobilized them.

Serum NAbs against SARS-CoV-2 were detectable following both regimens, displaying broad-spectrum neutralizing activity against prototype SARS-CoV-2 (WT) and VOCs (Beta, Delta, BA.1, BA.5, and XBB.1), although NAbs against BA.5 and XBB.1 were relatively feeble (Fig. 2c). Consistent with the BAb responses, the prime-boost regimen provoked higher NAb titers against all pseudoviruses compared to the prime-only regimen, with 0.95-fold to 9-fold increases for different variants. Furthermore, the live virus neutralization assay also demonstrated the superiority of the prime-boost regimen (Fig. 2d). There was a strong correlation between the 50% pseudovirus neutralization (pVNT_50_) titer and the 50% focus reduction neutralization (FRNT_50_) titer against WT and Delta strains (Fig. 2e), indicating that the pseudovirus neutralization assay reliably reflected the outcomes obtained through a live virus neutralization assay.

Vaccine-induced protective antibodies against pH1N1 were evaluated through hemagglutination inhibition (HAI) activity and neutralizing capacity. The WHO and European Committee for Medicinal Products considered HAI titers of 1:40 or greater are protective^27^. A single dose of AdC68-HATRBD vaccine achieved this threshold, and the HAI titers further increased after the second dose (Fig. 2f, left). Similar trends were observed for the NAb responses, where the prime-boost regimen outperformed the prime-only regimen (Fig. 2f, right).

**Fig. 3:**
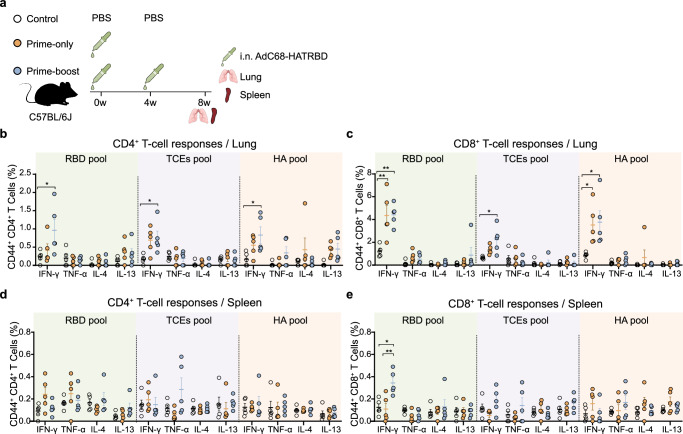
The prime-boost regimen triggers enhanced memory T-cell responses. **a** Scheme of experiments. The immunization schemes were identical to Fig. 2. Lung tissues and spleens were harvested at 8 week post-vaccination. **b**, **c** Frequencies of pulmonary CD4^+^ T cells (**b**) and CD8^+^ T cells (**c**) producing cytokines following re-stimulation with peptide pools for RBD, TCEs, and HA. **d**, **e** Frequencies of splenic CD4^+^ T cells (**d**) and CD8^+^ T cells (**e**) producing cytokines following re-stimulation with peptide pools for RBD, TCEs, and HA. All data are represented as mean ± SEM and analyzed by one-way ANOVA with Tukey correction. *P ≤ 0.05, **P ≤ 0.01.

We also valuated the ability of sera to neutralize AdC68 vector and found that AdC68-NAbs remained at baseline levels after i.n. priming but increased significantly after boosting (Fig. 3g). To further clarify the effect of pre-existing vector immunity on the efficacy of AdC68-HATRBD vaccine, mice were injected with AdC68-empty to induce vector-specific immunity, followed by i.n. immunization with two doses of AdC68-HATRBD (Supplementary Fig. 2a). Given the particularly high seroprevalence of human adenovirus serotype 5 (AdHu5) in humans^28^, the impact of pre-existing immunity to AdHu5 was evaluated using another set of mice pre-exposed to this virus. Strong BAb responses were observed in all vaccinated mice but not in unvaccinated controls (Supplementary Fig. 2b). When compared to non-exposed controls, mice pre-exposed to AdC68 or AdHu5 exhibited no reduction in the production of RBD- or HA-specific antibodies, suggesting that the antibody responses remained unaffected by pre-existing adenovirus immunity.

Overall, a single dose of AdC68-HATRBD stimulated modest humoral immune responses against SARS-CoV-2 and pH1N1, which were enhanced to high levels by a homologous booster.

### The prime-boost regimen triggers enhanced memory T-cell responses

Given the importance of potent cellular immune responses against SARA-CoV-2 and influenza virus for controlling and clearing the viruses^29,30^, we evaluated memory T-cell responses in mice following the prime-only and prime-boost regimens (Fig. 3a). Lymphocytes isolated from lung tissues were analyzed by intracellular cytokine staining assays using three sets of peptide pools: (i) an RBD peptide pool derived from BA.5, (ii) a TCEs peptide pool (Supplementary Table 1), and (iii) an HA peptide pool derived from pH1N1. The gating strategy for capturing the frequencies of T-cell subsets is shown in Supplementary Fig. 3. At 8 wpv, significant IFN-γ-producing CD4^+^ T-cell responses specific for RBD, TCEs, and HA were observed in mice receiving the prime-boost regimen rather than the prime-only regimen (Fig. 3b). Concurrently, there was a notable increase in memory IFN-γ^+^CD8^+^ T cells upon re-stimulation with peptide pools for RBD, TCEs, or HA in the prime-boost group compared to control group (Fig. 3c). The prime-only regimen also developed memory IFN-γ^+^CD8^+^ T-cell responses comparable to the prime-boost regimen. A parallel experiment was performed to evaluate cellular immunity in the spleens. The i.n. immunization with AdC68-HATRBD induced mild splenic memory T-cell responses, with only modest activation of RBD-specific IFN-γ^+^CD8^+^ T cells in prime-boost group (Fig. 3d, e).

Overall, i.n. immunization with AdC68-HATRBD drove antigen-specific T-cell immunity primarily in the lungs. A single dose of AdC68-HATRBD induced robust CD8^+^ but not CD4^+^ T-cell response, whereas the homologous i.n. booster augmented pH1N1- and SARS-CoV-2-specific cellular immunity, resulting in the simultaneous activation of CD4^+^ and CD8^+^ T cells. The T-cell activation was markedly skewed toward Th1 phenotype characterized by prominent IFN-γ secretion.

### Intranasal vaccination with AdC68-HATRBD protects K18-hACE2 mice from BA.5.2 challenge

**Fig. 4:**
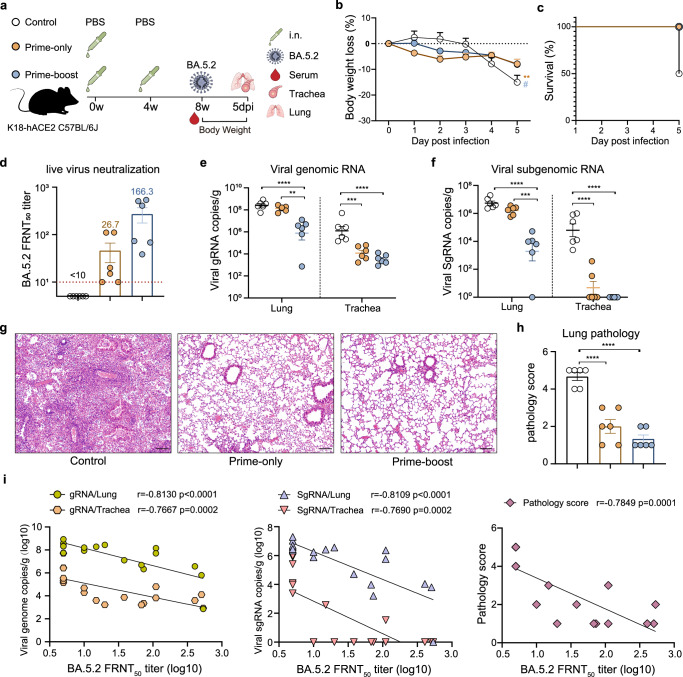
Intranasal vaccination with AdC68-HATRBD protects K18-hACE2 mice from BA.5.2 challenge. **a** Scheme of vaccination and challenge. K18-hACE2 mice (*n* = 6 per group) were immunized intranasally with 5 × 10^7^ IFU of AdC68-HATRBD using prime-only or prime-boost regimen. The control group received a placebo (PBS). At 8 weeks post-vaccination, mice were challenged with BA.5.2 and monitored for clinical signs and weight loss. Mouse serum was obtained to test for the neutralizing activity against BA.5.2 1 day prior to challenge. At 5 day post-infection, mice were euthanized, and tracheas and lung tissues were collected. **b**, **c** Percent weight loss (**b**) and percent survival (**c**) over 5 days post-infection. **d** Serum BA.5.2 neutralizing antibody titers prior to challenge. The red dashed line represents the lower limit of detection. **e**, **f** SARS-CoV-2 viral burden in the lungs and tracheas, measured by copies of the viral gRNA (**e**) and sgRNA (**f**). **g** Hematoxylin and eosin staining of lung sections from K18-hACE2 mice at 5 day post-infection. **h** Histopathological severity scoring was evaluated according to the pathological changes outlined in the methods section. **i** Spearman’s correlations of FRNT_50_ titers with viral gRNA copies (left), viral SgRNA copies (middle), and lung pathology scores (right). Each dot represents one animal. Values of geometric mean titer (GMT) are displayed in (**d**). Data are represented as GMT ± SD (**d**) or mean ± SEM (**b**, **c**, **e**, **f**, and **h**) and analyzed by one-way ANOVA with Tukey correction. In (**b**), asterisks (*) and pound signs (#) indicate the statistical significance level of control group compared with prime-only group and prime-only group, respectively, at 5 dpi. *^,#^*P* ≤ 0.05, **P ≤ 0.01, ***P ≤ 0.001, and ****P ≤ 0.0001. Scale bar, 50 μm.

We next evaluate the protective capacity of AdC68-HATRBD in a SARS-CoV-2 challenge model. K18-hACE2 transgenic mice were intranasally vaccinated with 5 × 10^7^ IFU of AdC68-HATRBD following a prime-only or prime-boost regimen and subsequently challenged with 1 × 10^5^ focus-forming units (FFU) of BA.5.2 at 8 wpv (Fig. 4a). PBS-immunized mice lost >15% of their body weight, and three of six mice succumbed to infection by 5 days post-infection (dpi) (Fig. 4b, c). In contrast, the AdC68-HATRBD-immunized mice exhibited minor weight loss and survived until the end of the experiment, regardless of the vaccination regimen. Both prime-only and prime-boost regimens induced significant NAbs against live BA.5.2 (Fig. 4d). In line with the NAb responses observed in wild-type C57BL/6 J mice, the prime-boost regimen induced significantly higher BA.5.2-NAbs in K18-hACE2 transgenic mice, with a 5.2-fold increase over the prime-only regimen.

At 5 dpi, the viral burden in the lungs and tracheas was assessed by quantifying the genomic RNA (gRNA) and subgenomic RNA (SgRNA) copies. The viral gRNA load in the lungs was considerably lower in the prime-boost group than control group, but not in the prime-only group. The gRNA reduction was also observed in the tracheas of all vaccinated mice (Fig. 4e). Consistent with the gRNA burden, the prime-only regimen lowered sgRNA copy number only in the tracheas, while the prime-boost regimen decreased sgRNA copy number in the lungs and tracheas (Fig. 4f). Histological analyses of the lung sections revealed significant acute inflammation in the control group (Fig. 4g, h). In contrast, mice immunized with the AdC68-HATRBD using the prime-only and prime-boost regimens had low overall pathology scores. Spearman’s correlation analysis revealed that viral gRNA/sgRNA loads in the lungs and tracheas as well as lung pathology scores were highly negatively related to the serum FRNT50 titers (Fig. 4i), demonstrating that the NAb level is an important predictor of vaccine efficacy.

Collectively, these results indicated that i.n. immunization with AdC68-HATRBD protected K18-hACE2 mice from the lethal BA.5.2 challenge. A homologous booster was likely to provide better protection against BA.5.2 infection in both the lungs and tracheas.

### Intranasal vaccination with AdC68-HATRBD protects against a lethal pH1N1 challenge

**Fig. 5:**
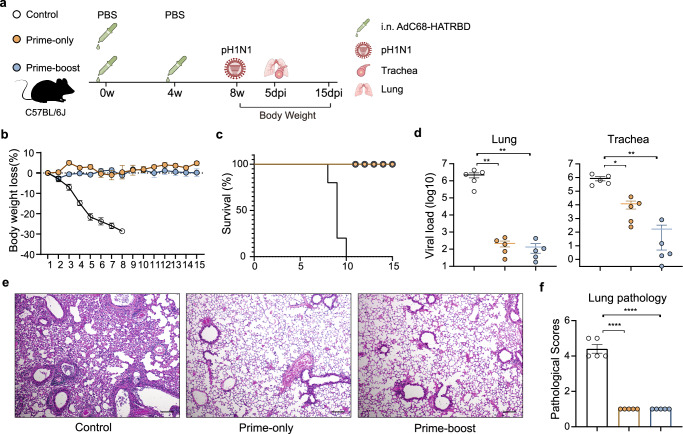
Intranasal vaccination with AdC68-HATRBD protects against a lethal pH1N1 challenge. **a** Scheme of vaccination and challenge. The immunization schemes were identical to Fig. 2. At 8 weeks post-vaccination, all mice were challenged with 10 × LD50 of pH1N1 virus. After the challenge, half of mice (*n* = 5 per group) were euthanized at 5 day post-infection with collection of tracheal and lung tissues, and the other half (*n* = 5 per group) were monitored for body weights and survival rates for 15 days. **b**, **c** Percent weight loss (**b**) and percent survival (**c**) over 15 days post-infection. **d** pH1N1 viral loads in mouse lungs (left) and tracheas (right) at 5 day post-infection. **e** Hematoxylin and eosin staining of lung sections at 5 day post-infection. **f** Pathology scores of the lungs at 5 day post-infection. Each dot represents one animal. Data are presented as mean ± SEM and analyzed by one-way ANOVA with Tukey correction. *P ≤ 0.05, **P ≤ 0.01, and ****P ≤ 0.0001. Scale bar, 50 μm.

We also assessed the ability of AdC68-HATRBD to protect mice from a lethal pH1N1 challenge. C57BL/6 J mice were immunized with AdC68-HATRBD using prime-only or prime-boost regimens and challenged with pH1N1 at 10 times the median lethal dose (LD50) at 8 wpv (Fig. 5a). PBS-immunized mice continuously lost weight and showed 100% mortality after challenge (Fig. 5b, c). In stark contrast, AdC68-HATRBD vaccine in prime-only and prime-boost regimens provided 100% protection against death and weight loss following a lethal pH1N1 challenge. Furthermore, vaccinated mice showed significantly reduced viral loads in the lungs and tracheas compared to the control group, regardless of the vaccination regimen (Fig. 5d). The tracheal viral load in the prime-boost group was 2.2 log10, which was 71-fold lower than in the prime-only group, although this decrease was not statistically significant. Histopathological analysis of lung sections at day 5 post-challenge revealed marked pathological changes in the control group, including bronchitis, bronchiolitis, and inflammatory cell infiltrates. In contrast, all vaccinated mice showed minimal histopathological changes (Fig. 5e). The overall lung pathological scores of the vaccinated mice in the prime-only or prime-boost regimens were comparable and significantly lower than those in the control group (Fig. 5f). These results demonstrate that i.n. immunization with AdC68-HATRBD protects mice from a lethal pH1N1 challenge. A homologous booster would confer more robust protection against BA.5.2 infection in the tracheas.

### **Heterologous AdC68-HATRBD booster reinforces and broadens the humoral immunity of licensed SARS-COV-2 vaccine**

**Fig. 6:**
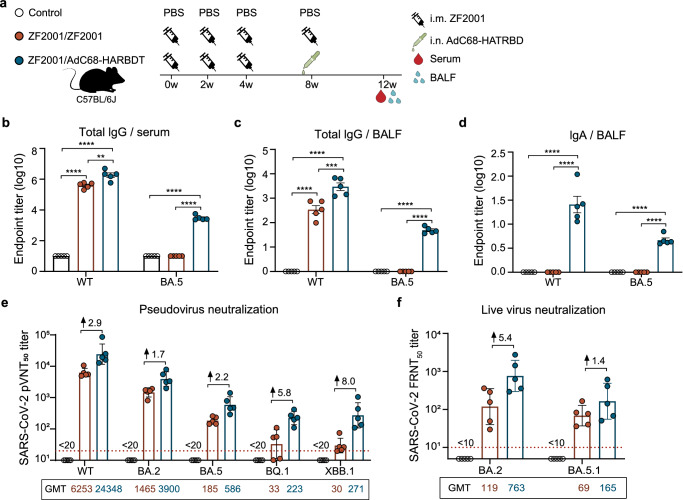
Heterologous AdC68-HATRBD booster reinforces and broadens the humoral immunity of licensed SARS-COV-2 vaccine. **a** Scheme of experiments. Mice were administrated three doses of 5 μg ZF2001 by i.m. route. After 4 weeks post-last vaccination, half of mice (*n* = 10 per group) received a homologous booster with 5 μg of ZF2001, the other half received a heterogenous i.n. booster with 5 × 10^7^ IFU of AdC68-HATRBD. Serum and BALF were harvested at 12 weeks post-vaccination. **b** Serum RBD-specific lgG antibody responses to wild-type SARS-CoV-2 (WT) and BA.5. **c**, **d** BALF RBD-specific lgG (**c**) and lgA (**d**) antibody responses to WT and BA.5. **e** Serum pseudovirus neutralizing antibody responses against WT and Omicron variants. **f** Serum live virus neutralizing antibody responses. Each dot represents data from two mice. In (**e**) and (**f**), values of geometric mean titer (GMT) are displayed, the red dashed line represents the lower limit of detection, and the numbers after the up arrow indicate the median fold increase in antibody titer induced by heterogenous vaccination compared with homologous vaccination. Data are presented as mean ± SEM (**b**–**d**) or GMT ± SD (**e**, **f**). Significance was assessed by one-way ANOVA with Tukey correction. **P ≤ 0.01, ***P ≤ 0.001, ****P ≤ 0.0001.

Considering the widespread pre-existing immunity to SARS-CoV-2 and influenza virus acquired from infection or vaccination in humans, we further explored the potential of sequential immunization with licensed vaccines followed by our combination vaccine. First, we investigated whether AdC68-HATRBD could trigger more ideal immune responses as a heterologous booster after licensed COVID-19 vaccine priming. Three doses of the ZF2001 vaccine administered intramuscularly as primary immunization have been widely used in China and many other countries^31^. Owing to its safety and efficacy, this vaccine was also approved for booster immunization in China on December 13, 2022^32^. We compared the immunogenicity of heterologous booster AdC68-HATRBD with that of homologous booster ZF2001 in mice previously vaccinated with ZF2001 (Fig. 6a). Homologous ZF2001/ZF2001 regimen exhibited high levels of WT-specific IgG but undetectable BA.5-specific IgG in the serum and BALF (Fig. 6b, c). In comparison, the heterologous ZF2001/AdC68-HATRBD regimen induced more robust IgG responses against WT and BA.5 RBD. The BALF IgA specific for WT and BA.5 were induced by the heterologous ZF2001/AdC68-HATRBD regimen, but not by the homologous ZF2001/ZF2001 regimen (Fig. 6d).

Subsequently, the ability of serum to neutralize WT and Omicron strains was measured. As previously shown^33^, the homologous ZF2001/ZF2001 vaccination induced high titers of NAbs against WT and BA.2 strains, but exhibited minimal neutralizing activity against BA.5, BQ.1, and XBB.1 (Fig. 6e). Compared with the homologous ZF2001/ZF2001 group, the heterologous ZF2001/AdC68-HATRBD group showed stronger neutralizing activity, with 2.9-fold, 1.7-fold, 2.2-fold, 5.8-fold, and 8.0-fold increases in pVNT_50_ titers against WT, BA.2, BA.5, BQ.1, and XBB.1, respectively. Consistent with the pVNT_50_ results, the NAb titers against live BA.2 and BA.5.1 were higher in the heterologous group than in the homologous group (Fig. 6f). These results indicated that the heterologous ZF2001/AdC68-HATRBD regimen was superior in improving humoral immunity, despite a weaker increase of Omicron-NAb than WT-NAb, regardless of the vaccine regimen.

Altogether, compared to a homologous booster, the heterologous booster with AdC68-HATRBD increased the magnitude and breadth of antibody responses, inducing mucosal lgA and broad-spectrum NAbs.

### Heterologous AdC68-HATRBD booster enhances the humoral and cell-mediated immunity of licensed influenza vaccine

**Fig. 7:**
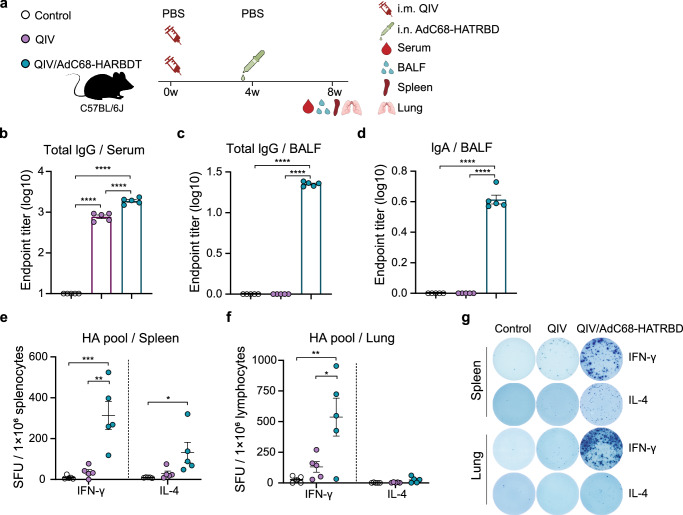
Heterologous AdC68-HATRBD booster enhances the humoral and cell-mediated immunity of licensed influenza vaccine. **a** Scheme of experiments. Mice (*n* = 5 per group) were intramuscularly immunized with 3 μg of QIV. Four weeks later, the prime-boost group received a heterogenous i.n. booster with 5 × 10^7^ IFU of AdC68-HATRBD. Eight weeks post-vaccination, serum, BALF, spleens, and lungs were collected. **b** Serum pH1N1 HA-specific lgG antibody titers. **c,** **d** BAFL pH1N1 HA-specific lgG (**c**) and lgA (**d**) antibody titers. **e**, **f** IL-4- and IFN-γ-secreting T cells specific for HA pool in the spleens (**f**) and lungs (**g**) were measured by ELISPOT assay. **g** Representative ELISpot showing HA-specific cytokines-secreting T cells. Each dot represents one animal. Data are presented as mean ± SEM. Asterisks indicate significant difference between groups determined using one-way ANOVA with Tukey correction. *P ≤ 0.05, **P ≤ 0.01, ***P ≤ 0.001, ****P ≤ 0.0001.

We assessed the immunogenicity of the combination vaccine AdC68-HATRBD as a booster for the licensed influenza vaccine. Mice were immunized using a prime-only or prime-boost regimen, with the former receiving a single dose of a quadrivalent inactivated influenza vaccine (QIV) intramuscularly and the latter receiving i.m. QIV followed by i.n. AdC68-HATRBD (Fig. 7a). Serum and BALF were collected at 8 wpv. HA-specific NAbs were detectable in the serum but not in the BAFL of QIV-immunized mice (Fig. 7b–d). The AdC68-HATRBD booster dramatically improved QIV-induced antibody responses, with increased serum IgG levels and the induction of BALF IgA and IgG.

We further assessed cellular immune response in the spleens and lungs. The numbers of IL-4- and IFN-γ-secreting T cells specific for the HA pool were evaluated by enzyme-linked immunospot (ELISpot) assay. A single dose of QIV was insufficient to activate splenic cellular immunity until AdC68-HATRBD was boosted (Fig. 7e–g). More specifically, compared with a single QIV vaccine dose, the AdC68-HATRBD booster elicited a 7.7-fold and 3.5-fold increase in splenic HA-specific IFN-γ and IL-4 responses, respectively. For pulmonary cellular immunity, the AdC68-HATRBD booster significantly rescued the low cellular response arising from QIV priming, activating IFN-γ responses.

Overall, the weak immunogenicity of i.m. QIV can be enhanced by heterologous booster AdC68-HATRBD, resulting in robust mucosal and systemic immune responses, including antibodies and cellular immunity.

## Discussion

To date, few studies have focused on developing a combined influenza/COVID-19 vaccine. Bao et al. found that co-inoculation with PiCoVacc (an inactivated SARS-CoV-2 vaccine, Sinovac Biotech Ltd) and an inactivated influenza vaccine (Sinovac Biotech Ltd) provoked NAbs against SARS-CoV-2 and H1N1 and completely protected K18-hACE2 mice from H1N1 and ancestral SARS-CoV-2 infections^34^. Two doses of i.m. immunization with an mRNA-based combination vaccine containing HA and RBD protected mice against co-infection with H1N1 and the SARS-CoV-2 Alpha and Delta variants^35^. Insertion of RBD fused with the conserved stalk of H7N9 HA into an adenoviral vector yielded a combination vaccine that protected against lethal SARS-CoV-2 and H7N9 challenges^36^. Influenza viral vectors carrying the SARS-CoV-2 RBD exhibited strong protective immunity against H1N1 and SARS-CoV-2^37,38^. Our study supports the effectiveness of the combination vaccine alone in preventing COVID-19 and influenza. Unlike the aforementioned studies, which were limited to ancestral RBD monomer or trimer, our study utilized two RBD heterodimers (a Beta-Alpha chimeric dimer and an Omicron-Delta chimeric dimer). In our study, AdC68-HATRBD, which carries two RBD heterodimers and conserved T-cell epitopes, induced broad-spectrum antibodies and cellular responses against SARS-CoV-2 and its variants.

With a low seroprevalence in humans^19,39^, AdC68 can induce strong adaptive immune responses against encoded antigens^40–42^, making it an attractive vaccine platform. However, the clinical use of adenovirus-based vaccines raises an important concern regarding the development of vector-specific immunity in vaccinated recipients, which may limit its use for boosting. In our study, we found that RBD- and HA-specific antibody responses induced by i.n. immunization with AdC68-HATRBD were not affected by pre-existing adenovirus immunity. Not coincidentally, previous studies have demonstrated that mucosal vaccination can bypass pre-existing immunity to adenovirus vector, thereby inducing potent immune responses specific for antigen^43,44^. In addition to varying immunization routes, numerous approaches are being extensively investigated to overcome adenoviral vector immunity. For example, covalent modification of adenoviral vectors using polymers such as polyethylene glycol and the macromolecular polysaccharide mannan can be effective in overcoming high-titer adenovirus-NAbs while retaining biological activity^45,46^. Encapsulation of adenoviral vectors with inert polymers, liposomes, and alginate microspheres has been shown to evade the vector-specific immune response^47–49^. Heterologous prime-boost immunization is also one way to address this problem^50,51^, as shown in Figs. 6 and 7.

A heterologous prime-boost regimen has proven to be superior to a homologous regimen in promoting antibody responses^52,53^, owing to an elevated frequency of switched and activated antigen-specific memory B cells^54^. In addition to heterologous combinations of vaccines, the combination of i.m. and i.n. immunization routes may also contribute to improved protective immunity, as demonstrated in our previous work^55^. In the context of the widespread use of COVID-19 and influenza vaccines, it is essential to evaluate combination vaccine candidates not only as standalone modalities but also with pre-existing immunity established by prior vaccination. Our work further demonstrates that the i.n. combination vaccine significantly enhances the protective immunity induced by licensed i.m. COVID-19 and influenza vaccines as a heterologous booster.

Real-world studies on heterologous vaccine schemes have primarily focused on the prevention and control of SARS-CoV-2 infections. In their interim guidance on Nov 10, 2023, WHO makes the following recommendations for heterologous COVID-19 vaccination schedules: countries implementing inactivated vaccines for initial doses may consider using vectored or mRNA vaccines for booster doses; countries implementing vectored vaccines for initial doses may consider using mRNA or protein subunit vaccines for booster doses; countries implementing mRNA vaccines for initial doses may consider using vectored or protein subunit vaccines for booster doses^5^. Numerous clinical studies have demonstrated that among individuals received two doses of inactivated COVID-19 vaccines, boosting with adenovirus vectored or mRNA vaccines provoked considerably stronger immune responses than in those boosted with inactivated vaccine^56,57^. A phase 2 trial comparing seven COVID-19 vaccines as a third booster showed an overall increase in reactogenicity of three vaccines: mRNA-1273 after ChAdOx1-S/ChAdOx1-S or BNT162b2/BNT162b2; and ChAdOx1-S and Ad26.COV2.S after BNT162b2/BNT162b2^58^. Obviously, our AdC68-HATRBD may be considered a booster dose for those who have received an inactivated, protein subunit or mRNA vaccine, in addition to being used in a one- or two-dose initial vaccination schedule. Noteworthy, following ChAdOx1-S/ChAdOx1-S, ChAdOx1-S did not enhance cellular responses, and antibody responses, although improved, were inferior to those of mRNA or protein subunit vaccines^58^. One of the reasons might be that the vector-specific immunity induced by initial doses impaired the immune responses to the homologous booster. The ChAdOx1-S vaccine is administered by intramuscular injection, a delivery route that cannot bypass pre-existing adenovirus immunity. Our study revealed that the antibody responses induced by i.n. immunization with AdC68-HATRBD was not hindered by pre-existing vector immunity in a mouse model, but the extent to which the results of immune responses in mice predict human outcomes of vaccination remains uncertain.

Next-generation vaccine is a promising strategy for combating the continued emergence of SARS-CoV-2 variants. However, immune imprinting may pose a challenge to the effectiveness of vaccination against variants. The immune response to Omicron subvariants could be compromised by immune imprinting in individuals who already have immunity against prototype or prototype-like strains through infection or vaccination^59,60^. Although booster vaccination with the prototype/BA.5 bivalent mRNA vaccine enhanced the antibody response to the Omicron subvariants, the neutralizing capacity against the Omicron subvariants remained much lower than against the prototype strain^61^. The immune imprinting phenomenon was also observed in our study, i.e., mice primed with ZF2001 and then boosted with AdC68-HATRBD maintained higher antibody titers against the prototype strain than Omicron subvariants (Fig. 6). In further study, we should probably discard prototype or prototype-like immunogens from the COVID-19 vaccine components to minimize the adverse effects of immune imprinting. For example, updating our AdC68-HATRBD vaccine by replacing the two chimeric RBD-dimers with the sequence from circulating Omicron subvariants such as XBB.1, EG.5, BA.2.86, and JN.1.

To control respiratory viral infections, mucosal immune responses at the site of infection, including NAbs, IgA, IgG, and virus-specific T cells, are critical^18,62^. Systemic IgG is effective in targeting viruses in the lungs but is usually much less so in the upper respiratory tract^63^. IgA is the hallmark of mucosal immunity, which hinders viral entry into the host cells by neutralizing viruses on mucosal surfaces, thereby establishing a first line of defense^64^. In our study, similar to other licensed i.m. vaccines^65,66^, mucosal IgA was poorly induced when vaccinated with only ZF2001 or QIV vaccines but remarkably activated after AdC68-HATRBD boosting, thus compensating for the shortcomings of licensed vaccines in inducing mucosal immunity.

Conventional SARS-CoV-2 and influenza vaccines based on inactivated virions or viral protein fragments, such as PiCoVacc, BBIBP-CorV, and NVX-CoV2373, are effective in inducing antibody responses, but not T-cell responses, particularly CD8^+^ T cells^67–70^. Next-generation vaccines based on mRNA or viral vectors, such as BNT162b2, mRNA-1273, Ad26.COV2.S, and ChAdOx1-S, can simultaneously induce robust humoral and cellular immune responses as they mimic viral infection by expressing antigens after immunization^71^. Indeed, AdC68-HATRBD but not QIV vaccine, elicited robust SARS-CoV-2- and influenza-specific Th1-biased CD4^+^ and CD8^+^ T cells. Notably, these responses were observed primarily in the lungs but not spleens. The weakness of i.n. AdC68-HATRBD to activate splenic T cells was altered in influenza vaccine-primed mice, although the licensed i.m. influenza vaccine alone did not induce a splenic cellular immune response. A study of T cell immunity in different human organs suggested that the lungs contained markedly higher frequencies of influenza- and respiratory syncytial virus-specific CD8^+^ T cells than the circulation^72^. Knudson et al. further demonstrated dramatic differences in the localization of T cell responses within the lung parenchyma between respiratory and systemic viruses, that is, most T cells are localized to the pulmonary vasculature following i.n. infection with a systemic pathogen, whereas T cells are primarily localized to the lung tissue (also known as lung tissue-resident memory T cells [T_RM_]) following a respiratory viral infection^73^. Given that the adenoviral vector AdC68 is also a respiratory virus, it is no wonder that i.n. immunization with AdC68-HATRBD would provoke primarily pulmonary T-cell responses, and it is logical to assume that a large number of T_RM_ cells are included in these antigen-specific T cells. Our recent study showed that an AdC68-based COVID-19 vaccine mounted SARS-CoV-2-specific lung T_RM_ cell responses through i.n. rather than i.m. immunization^55^.

Unlike antibody responses that are susceptible to viral mutations, T-cell immunity remains largely unaffected. Thus far, evidence indicates that highly infectious Omicron variants evade NAbs induced by vaccination or infection with other variants, thereby greatly increasing the prevalence of breakthrough infection. However, the vast majority of T cell responses to Omicron are preserved, which may contribute to the reduced clinical severity^74,75^. The same is true for the anti-influenza immune response^76,77^. The potent induction of pulmonary SARS-CoV-2- and influenza-specific T cells by AdC68-HATRBD may be beneficial for protection against circulating strains and potential future variants.

In conclusion, i.n. immunization with AdC68-HATRBD, which is cost-effective and easy to implement in pandemic settings, is immunogenic and protective against SARS-CoV-2 and influenza in mice. Maintaining this vaccine candidate as part of a heterologous regimen with the licensed SARS-CoV-2 and influenza vaccines may be a promising strategy to improve adaptive immune responses induced by primary vaccination, resulting in comprehensive protective immunity.

## Methods

### Cells and viruses

HEK-293, 293 T, Huh7, MDCK, and VeroE6 cells were all cultured in growth media (Dulbecco’s modified Eagle medium, 10% fetal bovine serum, and antibiotics) and incubated at 37 °C and 5% CO_2_. SARS-CoV-2 wild-type SH01 strain (GenBank: MT121215), Delta, BA.2, BA.5.1, and BA.5.2 were obtained from the Chinese Center for Disease Control and Prevention after isolation from patients in Shanghai, China. The pH1N1 influenza virus was provided by the Shanghai Public Health Clinical Center of Fudan University (Shanghai, China). The pH1N1 virus was created using reverse genetics and contained six gene segments from the A/PR8 virus and HA and NA genes from A/Califomia/7/2009.

### **Mouse**

Six- to eight-week-old female C57BL/6 J and K18-hACE2 transgenic mice were purchased from Beijing Vital River Laboratory Animal Technology Co., Ltd. (Beijing, China) and GemPharmatech Co., Ltd. (Jiangsu, China), respectively. All experiments involving live SARS-CoV-2 were conducted in the animal biosafety level 3 (BSL-3) facilities of Fudan University following International Standard Operating Procedures. All influenza virus experiments were performed in the BSL-2 facilities of the Shanghai Public Health Clinical Center of Fudan University following the standard operating protocols approved by the Institutional Biosafety Committee at the Shanghai Public Health Clinical Center, Fudan University. All animal studies that did not involve pathogenic pathogens were performed in SPF facilities, with the approval of the Institutional Animal Care and Use Committee of Tianjin Medical University (Tianjin, China).

### Vaccine construction

The recombinant AdC68 vector was engineered by deleting the E1/E3 gene and replacing the E4-orf6/7-orf4 gene with AdHu5 equivalents. The Beta-Alpha chimeric SARS-CoV-2 RBD dimer consisted of one Beta RBD (spike protein residues 319–541, GenBank: MZ410518.1) and one Alpha RBD (S protein residues 319–541, GenBank: OM443028.1) connected in tandem repeats. Omicron-Delta chimeric SARS-CoV-2 RBD dimer comprises one Omicron RBD (spike protein residues 319–541, GenBank: OP315382.1) and one Delta RBD (S protein residues 319–541, GenBank: ON908696.1) connected in tandem repeats. The tandem conserved TCEs from ORF1, ORF3, and M proteins of the ancestral SARS-CoV-2 are shown in Supplementary Table 1. The full-length HA gene sequence was based on pH1N1 (GenBank: ACP41953.1). These codon-optimized genes encoding the aforementioned transgene were inserted into the ∆E1 region of the AdC68 vector under the transcriptional control of the human cytomegalovirus promoter and the SV-40 polyadenylation sequence.

AdC68-HATRBD was packaged, rescued, and amplified in HEK293 cells and subsequently purified by cesium chloride density gradient ultracentrifugation. The infectious units of recombinant adenoviruses were titrated on HEK293 cells using QuickTiter™ Adenovirus Titer Immunoassay Kit (Cell Biolabs, San Diego, CA, USA) following the manufacturer’s instructions.

### Immunization and challenge

Mice were anesthetized with intraperitoneal tribromoethanol and then intranasally vaccinated with 5 × 10^7^ IFU of AdC68-HATRBD in a 15 μl volume. In the heterologous vaccination experiments, 5 μg of ZF2001 or 3 μg of QIV through i.m. route was administered. The control group received a placebo (PBS). At the end point of experiments, mice were euthanized by cervical dislocation and the BALF, spleens, and/or lungs were harvested. In Figs. 2, 3, and 6, each dot represents data from two mice. That is to say, the specimens were mixed from two mice, including BALF, serum and lymphocytes. In other animal experiments, each point represents data from one mouse.

Challenge was carried out with 1 × 10^5^ FFU of BA.5.2 or 10 × LD_50_ of pH1N1 administered intranasally. Mice were monitored daily for clinical signs and weight loss, with 70% of their initial weight considered a humane endpoint. To measure viral loads, the trachea and half a fraction of lung tissue were harvested on day 5 post-infection and immediately frozen in dry ice and stored at −80 °C until processed. The other half of the lung tissue was fixed in 4% paraformaldehyde for hematoxylin-eosin staining.

### Western blot

HEK293 cells were transduced with 10^5^, 10^6^, or 10^7^ IFU of AdC68-HATRBD. AdC68-empty-transduced (10^7^ IFU) HEK293 cells were used as controls. Twenty-four hours post-infection, the transduced cells were harvested and boiled at 100 °C with 1 × reductive loading buffer for 15 min. Western blotting was performed using anti-SARS-CoV-2 RBD antibody (1:2000 dilution, 40592-T62; Sino Biological, Beijing, China) or anti-influenza A HA antibody (1:5000 dilution, 86001-RM01; Sino Biological, Beijing, China), followed by HRP-conjugated anti-rabbit IgG antibody (1:5000 dilution, ab6721; Abcam, Cambridge, UK). β-actin was selected as the internal control (1:5000 dilution, HRP-60008; Proteintech, Wuhan, China). Blots and gels derived from the same or side-by-side experiments were processed together. The un-cropped and unprocessed western blot images are provided as Supplementary Fig. 4.

### Enzyme linked immunosorbent assay (ELISA) for binding antibody measurements

The IgG and IgA antibody levels in mouse serum and BALF were measured by ELISA, as previously described^55^. Briefly, ELISA plates were coated overnight at 4 °C with RBD protein of WT or BA.5 strain or HA protein of pH1N1 (100 ng/well, Sino Biological, Beijing, China). For lgG detection, serum samples were threefold serially diluted with 1:400 starting dilutions, and BALF samples were twofold serially diluted with 1:5 starting dilutions. For IgA detection, BALF samples were twofold serially diluted using an undiluted stock solution as the starting dilutions. Following 2-h incubation at 37 °C, secondary antibodies were added: HRP-conjugated anti-mouse IgG antibody (1:100000 dilution, ab6789; Abcam, Cambridge, UK); HRP-conjugated anti-mouse IgA (1:5000 dilution, 1040-05; Southern Biotech, Birmingham, AL, USA). Plates were again incubated for 1 h at 37 °C, followed by the addition of TMB substrate (NCM Biotech, Suzhou, China). Sulfuric acid (2 M H_2_SO_4_) solution was used to stop the reaction. The optical density (OD)_450–630_ was recorded using a microplate reader (Tecan, Männedorf, Switzerland). The binding antibody endpoint titer was determined as the reciprocal of the highest serum dilution that yielded an absorbance greater or equal to 0.1 OD unit above the absorbance of the pre-immune samples.

### Production of SARS-CoV-2 pseudoviruses

SARS-CoV-2 pseudoviruses were produced as previously described^78,79^. Briefly, the backbone plasmid pNL4-3.Luc.R-E and pCAGGS-S-CΔ19 expressing spike of the WT strain or its variants were cotransfected into HEK 293 T cells by polyethyleneimine (Polysciences, Warrington, PA, USA). The spike amino acid sequences of WT strain and its variants were based on GISAID EPI_ISL_402124 (Wuhan/WIV04/2019), EPI_ISL_712096 (Beta variant), EPI_ISL_2029113 (Delta variant), EPI_ISL_6640916 (BA.1 variant), EPI_ISL_8515362 (BA.2 variant), EPI_ISL_13241867 (BA.5 variant), EPI_ISL_15812431 (BQ.1 variant), or EPI_ISL_14917761 (XBB.1 variant). The supernatant was collected 48 h post-transfection, filtered through 0.45 µm filters, and concentrated overnight with PEG 8000 at 4 °C.

Pseudovirus titration was performed based on HIV-1 p24 antigen quantification utilizing Lentivirus Quantitation Kit (Beijing Biodragon Immunotechnologies Co., Ltd, Beijing, China) following the manufacturer’s instructions; 1 × 10^8^ lentivirus particles per well were used for the pseudovirus neutralization test.

### SARS-CoV-2 pseudovirus neutralization assay

Huh7 cells were seeded at a density of 2.5 × 10^4^ cells per well in opaque 96-well flat-bottom plates. Twenty-four hours later, each serum sample was threefold serially diluted and mixed with an equal volume of pseudoviruses. The mixture was incubated at 37 °C for 1 h before adding to Huh7 cells. Relative luciferase activity (RLA) was measured after 48 h using the SteadyGlo Luciferase Assay System (Promega, Madison, WI, USA). The pVNT_50_ titer was calculated as the reciprocal of the highest serum dilution at which RLA was reduced by 50% compared to the RLA in virus control wells infected with pseudovirus in the absence of mouse serum. The lower limit of detection (LLD) of the neutralization assay was 20, and measurements below the LLD were assigned half the LLD.

### SARS-CoV-2 neutralization assay

Serum samples were threefold serially diluted in 96 well U-bottom plates. An equal volume of SARS-CoV-2 consisting of WT strain, Delta, BA.2, BA.5.1, or BA.5.2 was then added to the diluted serum. The serum-virus mixture was incubated at 37 °C for 1 h before transferring to 96-well plates with 4 × 10^4^ VeroE6 cells per well. Plates were incubated at 37 °C, 5% CO_2_ for 48 h, and fixed with 4% paraformaldehyde in PBS for 20 min. The plates were washed and incubated with rabbit anti-N antibody (1:2000 dilution, A18797; Abclonal, Wuhan, China), followed by incubation with peroxidase-conjugated anti-rabbit IgG (1:1000 dilution, AS014; Abclonal, Wuhan, China) and peroxidase substrate (Seracare, Milford, MA, USA). Virus-infected cell foci were counted using an ImmunoSpot microanalyzer (CTL, Shaker Heights, OH, USA). The FRNT_50_ titer was measured as the reciprocal of the highest serum dilution at which foci were reduced by 50% relative to the control wells infected with SARS-CoV-2 in the absence of mouse serum. The LLD of the neutralization assay was 10, and measurements below the LLD were assigned half the LLD.

### Influenza virus neutralization assay

The influenza virus-specific neutralization activity of mouse serum was analyzed using a micro-neutralization assay in MDCK cells. A two-fold serial dilution of serum samples was performed in a 96-well plate and incubated with 100 tissue culture infectious dose 50 (TCID50) of pH1N1 at 37 °C for 1 h. The MDCK culture media was then replaced with the mixture and incubated at 37 °C for 48 h. The culture supernatants were then cultured with same volume of 1% chicken red blood cells diluted in PBS and incubated for 20 min at room temperature. The neutralizing titer was the highest dilution of serum that inhibited virus-induced hemagglutination. The LLD of the neutralization assay was 10, and measurements below the LLD were assigned half the LLD.

### Haemagglutination-inhibition assay

Serum samples were treated with a receptor-destroying enzyme from Vibrio cholerae (Denka Seiken, Tokyo, Japan) at 37 °C overnight, followed by heat-inactivation at 56 °C for 30 min. Serial serum dilutions were mixed with four hemagglutination units of inactivated pH1N1 virus in 96 well V-bottom plates for 1 h. The 1% chicken red blood cells diluted in PBS were added to each well, and the wells were incubated for 15 min before analysis. The reciprocal of the highest serum dilution that prevented complete hemagglutination was considered the HAI titer. The LLD of the HAI assay was 10, and measurements below the LLD were assigned half the LLD.

### Adenovirus neutralization assay

As previously described, 5 × 10^4^ cells per well A549 were seeded in 96-well overnight. A threefold dilution series of the test sera were mixed with an equivalent volume of 1 × 10^7^ vp AdC68-Luc. After 2 h of incubation at 37 °C, the mixture was added to A549 cells. Two days later, RLA was measured. The NT50 titer was calculated as the reciprocal of the highest serum dilution at which RLA was reduced by 50% compared to the RLA in virus control wells infected with AdC68-Luc in the absence of mouse serum. The LLD of the neutralization assay was 10, and measurements below the LLD were assigned half the LLD.

### Flow cytometry

As previously described^40,80^, the mononuclear cells from lungs and spleens were isolated and stimulated with 2 µg/mL of the RBD, TCEs, or HA pool in the presence of GolgiPlug (BD Biosciences, San Jose, CA, USA) for 12 h. The RBD peptide pool contained 15-mers overlapping by 11 amino acids derived from the BA.5 variant. The TCEs peptide pool contained 24 epitope peptides (Supplementary Table 1). The HA peptide pool contained 15-mers overlapping by 11 amino acids derived from pH1N1. Upon stimulation, cells were blocked with Fc block (1:250 dilution, 14-0161-85; BD Biosciences, Franklin Lakes, NJ, USA) and simultaneously stained with LIVE/DEAD Fixable dye (Thermo Fisher Scientific, Waltham, MA, USA) at 4 °C for 30 min. Cells were then stained with four surface markers containing PerCP/Cyanine5.5-CD3ε (1:500 dilution, 100328; BioLegend, San Diego, CA, USA), APC-Cy7-CD4 (1:250 dilution, A15384; Thermo Fisher Scientific, Waltham, MA, USA), Alexa Fluor 700-CD8a (1:250 dilution, 100730; BioLegend, San Diego, CA, USA), and FITC-CD44 (1:250 dilution, 48-0621-80; Thermo Fisher Scientific, Waltham, MA, USA) for 30 min at 4 °C, followed by fixation and permeabilization using fixation/permeabilization solution (BD Biosciences, San Jose, CA, United States) following the manufacturer’s instruction. Fixed/permeabilized cells were incubated with a cocktail of fluorescently labeled cytokine antibodies containing APC-IFN-γ (1:250 dilution, 505810; BioLegend, San Diego, CA, USA), Brilliant Violet 421-TNF-α (1:500 dilution, 506328; BioLegend, San Diego, CA, USA), PE-IL-13 (1:250 dilution, 159403; BioLegend, San Diego, CA, USA), and PE-Cy7-IL-4 (1:250 dilution, 504118; BioLegend, San Diego, CA, USA) for 30 min at 4 °C. Finally, samples were fixed with 2% PFA for 30 min before the acquisition, then acquired on a BD LSRFortessa (BD Bioscience, San Jose, CA, USA) and analyzed using FlowJo v10.

### ELISpot assay

Single cells of spleens and lungs were isolated to determine the number of cytokines-producing cells using Mouse IFN-γ ELISpotBASIC kit and Mouse IL-4 ELISpotBASIC kit (MabTech, Nacka Strand, Sweden) according to the manufacturer’s instruction. Briefly, Multiscreen IP ELISpot plates (Millipore, Billerica, MA, USA) were coated with respective capture antibody overnight at 4 °C. After blocking and washing, 1 × 10^6^ cells and 2 µg/ml HA pool were added to each well of the plate, and incubated for 48 h at 37 °C with 5% CO_2_. Spot counts were performed using an ImmunoSpot microanalyzer (CTL, Shaker Heights, OH, USA). The results are presented as the number of spot-forming unit (SFU) per well.

### Viral load determination

The loads of SARS-CoV-2 and influenza virus in the tracheas and lungs were quantified using quantitative real-time-polymerase chain reaction from the RNA samples prepared as described above. For SARS-CoV-2, two sets of primers were used to detect the N gene of the viral genome and the E gene of sgRNA.

N Forward: 5′-GACCCCAAAATCAGCGAAAT-3′;

N Reverse: 5′-CTGGTTACTGCCAGTTGAATCTG-3;

N-probe: FAM-ACCCCGCATTACGTTTGGTGGACC-TAMRA;

E Forward: 5′-CGATCTCTTGTAGATCTGTTCTC-3′;

E Reverse: 5′-ATATTGCAGCAGTACGCACACA-3′;

E-probe: FAM-ACACTAGCCATCCTTACTGCGCTTCG-TAMRA.

SARS-CoV-2 viral loads were expressed on a log10 scale as viral copies per gram after calculation with a standard curve.

For influenza virus, the matrix (M) gene amplicon was quantified using the following primers:

M Forward: 5′-AAGACCAATCCTGTCACCTCTGA-3′;

M Reverse: 5′-CAAAGCGTCTACGCTGCAGTCC-3.

Ct values were normalized to the calibrator mouse gene GAPDH.

Genome was extracted from lungs and nasal turbinates using TIANamp Genomic DNA kit (Tiangen, Beijing, China). The genome load of AdC68-HATRBD was quantified by RT-qPCR using the Roche LightCycler® 480 system. The primer sequences to detect the hexon gene of AdC68 were:

Hexon Forward: 5′-GCCCTTCCACATCCAGGTGC-3′;

Hexon Reverse: 5′-CCATGGGGAAGAAGGTGGCG-3′;

AdC68-HATRBD load was expressed as viral copies per gram after calculation with a standard curve.

### Histopathology

PFA-fixed lung tissues were embedded in paraffin, sectioned into 4 µm-thick, and stained with hematoxylin and eosin. According to previous study^41^, histopathological changes were scored from 1 to 5 based on the degree of interalveolar edema, intra-alveolar hemorrhage, and neutrophil infiltration.

### Statistical analysis

All data are presented as means ± SEM unless otherwise stated, and asterisks and pound signs in the figures indicate statistical significance (*P < 0.05, **P < 0.01, ***P < 0.001, and ****P < 0.0001). All experimental data used to compare differences among groups were analyzed via one-way ANOVA with Tukey’s multiple comparisons test using GraphPad Prism 8 software (GraphPad Software, San Diego, CA, USA).

### Reporting summary

Further information on research design is available in the Nature Research Reporting Summary linked to this article.

### Supplementary information

Supplementary information

REPORTING SUMMARY

## Data Availability

The data that support the findings of this study are available from the corresponding author upon reasonable request.
