# Supplementary material for: An intranasal combination vaccine induces systemic and mucosal immunity against COVID-19 and influenza
Source: NPJ Vaccines. 2024 Mar 21;9:64. doi: 10.1038/s41541-024-00857-5 (PMC10954707; doi:10.1038/s41541-024-00857-5)
Supplement: Supplementary file 1 — Supplementary information [file 41541_2024_857_MOESM1_ESM.pdf]

Supplementary Information

Supplementary Figure 1

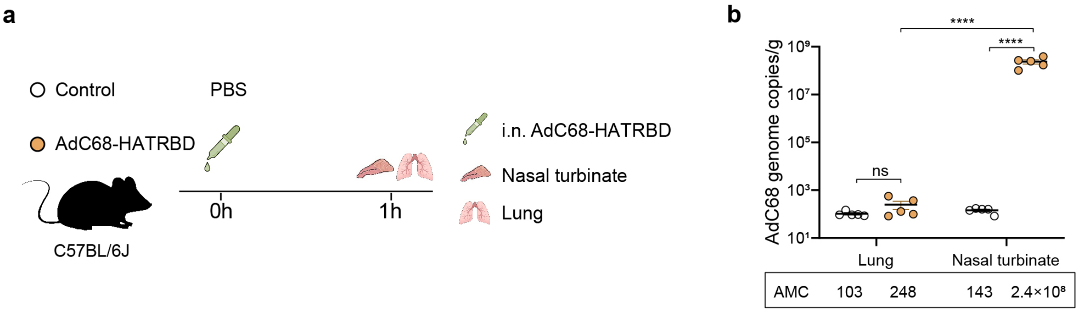

Supplementary Figure 1. After intranasal administration, the vaccine is primarily distributed in the upper respiratory tract but not the lungs.

a. Scheme of experiments. Mice (n = 5 per group) were intranasally administered with a single dose of 5×10^7 IFU of AdC68-HATRBD. The control group received a placebo (PBS). Lungs and nasal turbinates were collected at 1 hour post-vaccination. **b.** The copy number of AdC68 genome was detected by qPCR assay. Values of arithmetic mean copy number (AMC) were displayed in (b). Data are presented as mean ± SEM and analyzed by two-way ANOVA with Tukey correction. ns, no significant. ****$P \leq 0.0001$.

Supplementary Figure 2

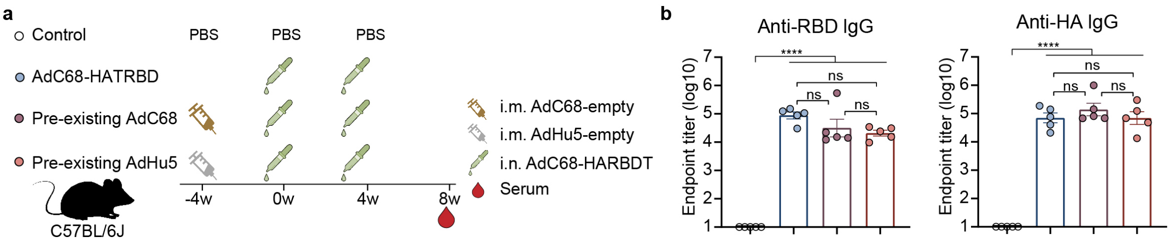

Supplementary Figure 2. The antibody responses were unaffected by pre-existing adenovirus immunity

a Scheme of experiments. Mice (n = 10 per group) were intramuscularly administered with a single dose of 5×10^7 IFU of AdC68-empty or AdHu5-empty. After 4 weeks, the pre-exposed and non-exposed mice received two doses, 4 weeks apart, of 5×10^7 IFU of AdC68-HATRBD via i.n. rout. The control group received a placebo (PBS). Serum was harvested at 8 week post-vaccination. **b** Binding antibody titers of the serum lgG specific to RBD (left) and HA (right), expressed in log10. Each dot represents data from two mice. Data are represented as mean ± SEM and analyzed by one-way ANOVA with Tukey correction. ns, no significant. ****$P \leq 0.0001$.

Supplementary Figure 3

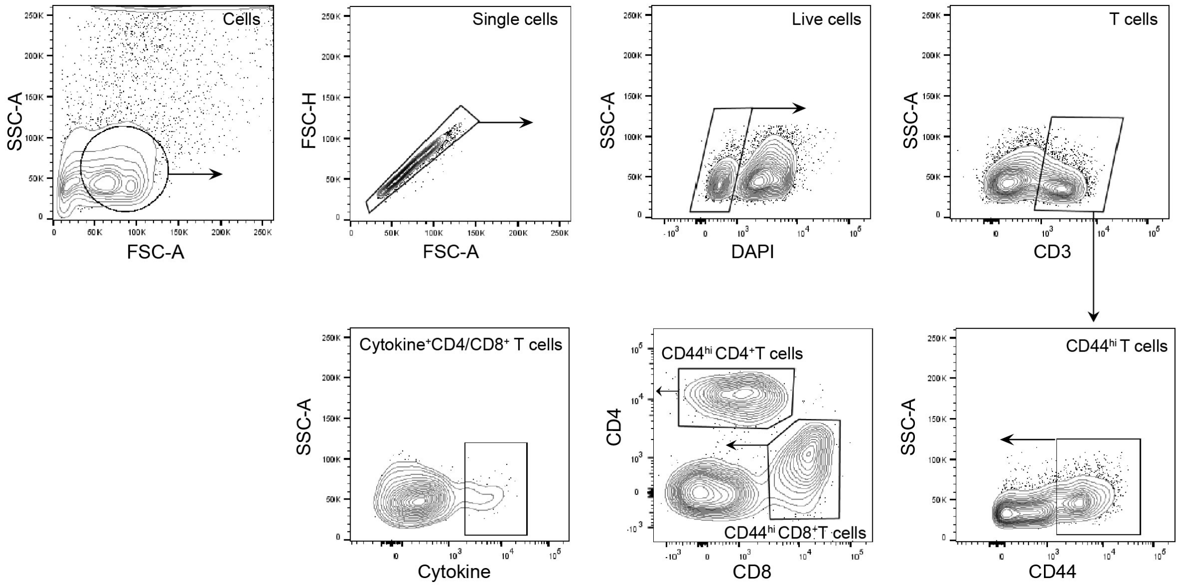

Supplementary Figure 3. Schematics of gating strategy for the analysis of T cell subpopulations by flow cytometry.

After gating for live lymphocytes in forward and side scatter, cell aggregates/doublets were ignored. T cells ($CD44^{hi}CD3^{+}$) were stratified into CD4 T cells ($CD4^{+}$) and CD8 T cells ($CD8^{+}$), and their subsets were analyzed for cytokine expression levels, including IFN-γ, TNF-α, IL-4, and IL-13.

Supplementary Figure 4

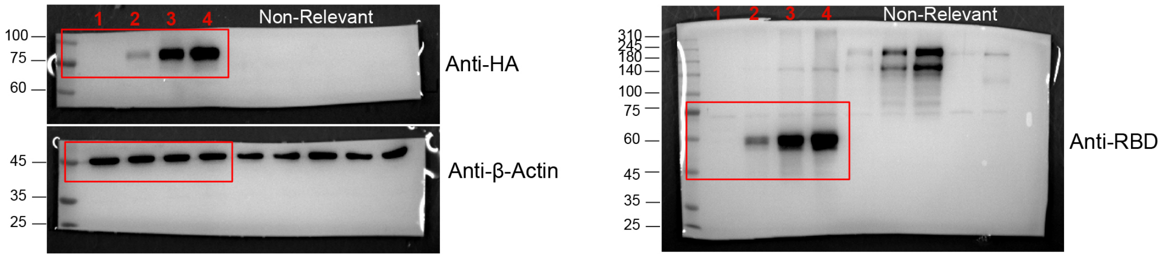

Supplementary Figure 4. Un-cropped and unprocessed images used to generate Fig. 1c, showing RBD dimers and HA in AdC68-HATRBD transduced HEK 293 cells.

Red boxes indicate the regions shown in Fig.1c. Other bands are not relevant to this paper.

Supplementary Table 1. Detailed information on tandem conserved T-cell epitopes (TCEs).

Peptides Name	The lengths of peptides	Peptides used for stimulations	Epitopes used in the vaccine	T cell epitopes in published studies	HLA restriction	Studies (References)
ORF1-1	10 aa	TTDPSFLGRY	TTDPSFLGRY	TTDPSFLGRY	A*01:01	[1-4]
ORF1-2	10 aa	CTDDNALAYY	CTDDNALAYY	CTDDNALAYY	A*01:01	[2,4,5]
ORF1-3	9 aa	YLITPVHVM	YLITPVHVM	YLITPVHVM	A*02:01	[2,6]
ORF1-4	9 aa	FYYVWKSYV	FYYVWKSYV	FYYVWKSYV	C*06:02	[2]
ORF1-5	9 aa	NSFSGYLKL	NSFSGYLKL	NSFSGYLKL	C*06:02	[2]
ORF3-1	9 aa	VYFLQSINF	VYFLQSINF	VYFLQSINF	A*24	[1-3]
ORF3-2	9 aa	ALSKGVHFV	ALSKGVHFV	ALSKGVHFV	A*02:01	[1-3,7]
ORF3-3	15 aa	CRSKNPLLYDANYFL	CRSKNPLLYDANYFLCWHTNCYDYCIPYNSVTSSI	NPLLYDANYFL	A*02:01	[2,8]
ORF3-4	15 aa	NPLLYDANYFLCWHT		LLYDANYFL	A*02:01	[2,4,5,7,8]
ORF3-5	15 aa	YDANYFLCWHTNCYD		LYDANYFLCW	B*57:01	[8]
ORF3-6	15 aa	YFLCWHTNCYDYCIP		FLCWHTNCY	A*01:01	[2,8]
ORF3-7	15 aa	WHTNCYDYCIPYNSV		FLCWHTNCYDYCIPY	DQB1*05:03	[9]
ORF3-8	15 aa	CYDYCIPYNSVTSSI		IPYNSVTSSI	A*24:02	[2,7,8]
M-1	15 aa	RLFARTRSMWSFNPE	RLFARTRSMWSFNPETNILLNVPLHGTILTRPLLESELVIGAVILRGHLRIAGHL	RLFARTRSMW	A*02:01	[7,8]
				RLFARTRSMWSFNPE	DRB1*14:01	[9,10]
M-2	15 aa	RTRSMWSFNPETNIL		RTRSMWSFNPETNILLNVPL	DRB1*01:01	[11]
				SMWSFNPET	A*02:01	[8]
M-3	15 aa	MWSFNPETNILLNVP		SFNPETNIL	B*08:01	[2,7,8]
M-4	15 aa	NPETNILLNVPLHGT		SFNPETNILLNVPLH	DRB1*01:02	[9,10]
M-5	15 aa	NILLNVPLHGTILTR		NVPLHGTIL	B*07:02	[7]
				VPLHGTILTR	A*03:01	[2,7]
M-6	15 aa	NVPLHGTILTR		NVPLHGTILTR	HLA class	[9,10]

		PLLE		PLLE	II	
M-7	15 aa	HGTILTRPLLESELV		GTILTRPLLESELVI	DRB1*13:01	[9,10]
M-8	15 aa	LTRPLLESELVIGAV		RPLLESELV	B*07:02	[7]
M-9	15 aa	LLESELVIGAVILRG		RPLLESELVIGAVIL	DQB1*02:02	[9]
M-10	15 aa	ELVIGAVILRGHLRI		GAVILRGHL	B*07:02	[7]
M-11	15 aa	GAVILRGHLRIAGHHL		AVILRGHL	B*08:01	[2]
				HLRIAGHHL	B*15:01	[2,7,8]

Supplementary references

1 Nelde, A. *et al.* SARS-CoV-2-derived peptides define heterologous and COVID-19-induced T cell recognition. *Nature immunology* **22**, 74-85, doi:10.1038/s41590-020-00808-x (2021).

2 Saini, S. K. *et al.* SARS-CoV-2 genome-wide T cell epitope mapping reveals immunodominance and substantial CD8(+) T cell activation in COVID-19 patients. *Science immunology* **6**, doi:10.1126/sciimmunol.abf7550 (2021).

3 Minervina, A. A. *et al.* SARS-CoV-2 antigen exposure history shapes phenotypes and specificity of memory CD8(+) T cells. *Nature immunology* **23**, 781-790, doi:10.1038/s41590-022-01184-4 (2022).

4 Lang-Meli, J. *et al.* SARS-CoV-2-specific T-cell epitope repertoire in convalescent and mRNA-vaccinated individuals. *Nature microbiology* **7**, 675-679, doi:10.1038/s41564-022-01106-y (2022).

5 Schulien, I. *et al.* Characterization of pre-existing and induced SARS-CoV-2-specific CD8(+) T cells. *Nature medicine* **27**, 78-85, doi:10.1038/s41591-020-01143-2 (2021).

6 Palatnik-de-Sousa, I. *et al.* A novel vaccine based on SARS-CoV-2 CD4(+) and CD8(+) T cell conserved epitopes from variants Alpha to Omicron. *Scientific reports* **12**, 16731, doi:10.1038/s41598-022-21207-2 (2022).

7 Sekine, T. *et al.* Robust T Cell Immunity in Convalescent Individuals with Asymptomatic or Mild COVID-19. *Cell* **183**, 158-168 e114, doi:10.1016/j.cell.2020.08.017 (2020).

8 Nathan, A. *et al.* Structure-guided T cell vaccine design for SARS-CoV-2 variants and sarbecoviruses. *Cell* **184**, 4401-4413 e4410, doi:10.1016/j.cell.2021.06.029 (2021).

9 Tarke, A. *et al.* Comprehensive analysis of T cell immunodominance and immunoprevalence of SARS-CoV-2 epitopes in COVID-19 cases. *Cell reports. Medicine* **2**, 100204, doi:10.1016/j.xcrm.2021.100204 (2021).

10 Heide, J. *et al.* Broadly directed SARS-CoV-2-specific CD4+ T cell response includes frequently detected peptide specificities within the membrane and nucleoprotein in patients with acute and resolved COVID-19. *PLoS pathogens* **17**, e1009842, doi:10.1371/journal.ppat.1009842 (2021).

11 Johansson, A. M. *et al.* Cross-reactive and mono-reactive SARS-CoV-2 CD4+ T cells in prepandemic and COVID-19 convalescent individuals. *PLoS pathogens* **17**, e1010203, doi:10.1371/journal.ppat.1010203 (2021).
