# Supplementary material for: An intranasal combination vaccine induces systemic and mucosal immunity against COVID-19 and influenza
Source: NPJ Vaccines. 2024 Mar 21;9:64. doi: 10.1038/s41541-024-00857-5 (PMC10954707; doi:10.1038/s41541-024-00857-5)
Supplement: Supplementary file 2 — REPORTING SUMMARY [file 41541_2024_857_MOESM2_ESM.pdf]

nature portfolio

Corresponding author(s): NPJVACCINES-03514

Last updated by author(s): Jan 23, 2024

Reporting Summary

Nature Portfolio wishes to improve the reproducibility of the work that we publish. This form provides structure for consistency and transparency in reporting. For further information on Nature Portfolio policies, see our Editorial Policies and the Editorial Policy Checklist.

Statistics

For all statistical analyses, confirm that the following items are present in the figure legend, table legend, main text, or Methods section.

n/a	Confirmed	
☐	☒	The exact sample size (*n*) for each experimental group/condition, given as a discrete number and unit of measurement
☐	☒	A statement on whether measurements were taken from distinct samples or whether the same sample was measured repeatedly
☐	☒	The statistical test(s) used AND whether they are one- or two-sided<br>*Only common tests should be described solely by name; describe more complex techniques in the Methods section.*
☐	☒	A description of all covariates tested
☐	☒	A description of any assumptions or corrections, such as tests of normality and adjustment for multiple comparisons
☐	☒	A full description of the statistical parameters including central tendency (e.g. means) or other basic estimates (e.g. regression coefficient) AND variation (e.g. standard deviation) or associated estimates of uncertainty (e.g. confidence intervals)
☐	☒	For null hypothesis testing, the test statistic (e.g. *F*, *t*, *r*) with confidence intervals, effect sizes, degrees of freedom and *P* value noted<br>*Give P values as exact values whenever suitable.*
☒	☐	For Bayesian analysis, information on the choice of priors and Markov chain Monte Carlo settings
☒	☐	For hierarchical and complex designs, identification of the appropriate level for tests and full reporting of outcomes
☐	☒	Estimates of effect sizes (e.g. Cohen's *d*, Pearson's *r*), indicating how they were calculated

Our web collection on statistics for biologists contains articles on many of the points above.

Software and code

Policy information about availability of computer code

Data collection	N/A
Data analysis	GraphPad Prism 8.0.1 (GraphPad Software)

For manuscripts utilizing custom algorithms or software that are central to the research but not yet described in published literature, software must be made available to editors and reviewers. We strongly encourage code deposition in a community repository (e.g. GitHub). See the Nature Portfolio guidelines for submitting code & software for further information.

Data

Policy information about availability of data

All manuscripts must include a data availability statement. This statement should provide the following information, where applicable:

- Accession codes, unique identifiers, or web links for publicly available datasets
- A description of any restrictions on data availability
- For clinical datasets or third party data, please ensure that the statement adheres to our policy

The authors declare that all other data supporting the findings of this study are available in the Article and its Supplementary Information and are also available from the corresponding author on request.

Research involving human participants, their data, or biological material

Policy information about studies with human participants or human data. See also policy information about sex, gender (identity/presentation), and sexual orientation and race, ethnicity and racism.

Reporting on sex and gender	N/A<br>N/A<br>N/A
Reporting on race, ethnicity, or other socially relevant groupings	N/A
Population characteristics	N/A
Recruitment	N/A
Ethics oversight	N/A

Note that full information on the approval of the study protocol must also be provided in the manuscript.

Field-specific reporting

Please select the one below that is the best fit for your research. If you are not sure, read the appropriate sections before making your selection.

☒ Life sciences ☐ Behavioural & social sciences ☐ Ecological, evolutionary & environmental sciences

For a reference copy of the document with all sections, see nature.com/documents/nr-reporting-summary-flat.pdf

Life sciences study design

All studies must disclose on these points even when the disclosure is negative.

Sample size	In order to use the minimal number based on ethical considerations and the cost of animals and housing, statistical analyses were performed to simulate the significance of the results. The following assumptions in the analysis of the data are reasonable based on experience in vaccine and gene therapy research. (1) Paired data analysis if different sets of animals are used for each group. (2) Gaussian distribution. (3) Equal standard deviations in the control and treatment/disease groups. (4) A two-tailed P value, which is the most conservative although the direction of the change is often predictable. Based on these assumptions, the goal is to determine the number of animals needed to achieve a significance of p <0.05 for various differences between the control group and the treatment/disease group with either 15% or 20% coefficient of variation (CV) for the group statistic. Based on above calculation, we set up 5-10 mice in each group to achieve a significance of p <0.05 for various differences between groups.between experimental groups.
Data exclusions	No data were excluded.
Replication	Reproducibility of in vitro, ex vivo and in vivo experiments was verified where possible by repeating the experiment on a different day with a unique batch of materials.
Randomization	For all mice experiments, the mice are randomized into cohorts of five by the animal house staff.
Blinding	For vaccine efficacy experiments, mice were immunized intranasally on days 0 and 28. Before challenge, mice undergoing procedure were blinded by a researcher from the lab not directly involved in the experiment or by the animal house staff. Mice were unblinded once all data had been procured and analyzed.<br>experiment or by the animal house staff. Mice were unblinded once all data had been procured and analyzed.

Behavioural & social sciences study design

All studies must disclose on these points even when the disclosure is negative.

Study description	N/A
Research sample	N/A
Sampling strategy	N/A
Data collection	N/A
Timing	N/A

Data exclusions	N/A
Non-participation	N/A
Randomization	N/A

Ecological, evolutionary & environmental sciences study design

All studies must disclose on these points even when the disclosure is negative.

Study description	N/A
Research sample	N/A
Sampling strategy	N/A
Data collection	N/A
Timing and spatial scale	N/A
Data exclusions	N/A
Reproducibility	N/A
Randomization	N/A
Blinding	N/A

Did the study involve field work? ☐ Yes ☒ No

Field work, collection and transport

Field conditions	*Describe the study conditions for field work, providing relevant parameters (e.g. temperature, rainfall).*
Location	*State the location of the sampling or experiment, providing relevant parameters (e.g. latitude and longitude, elevation, water depth).*
Access & import/export	*Describe the efforts you have made to access habitats and to collect and import/export your samples in a responsible manner and in compliance with local, national and international laws, noting any permits that were obtained (give the name of the issuing authority, the date of issue, and any identifying information).*
Disturbance	*Describe any disturbance caused by the study and how it was minimized.*

Reporting for specific materials, systems and methods

We require information from authors about some types of materials, experimental systems and methods used in many studies. Here, indicate whether each material, system or method listed is relevant to your study. If you are not sure if a list item applies to your research, read the appropriate section before selecting a response.

Materials & experimental systems

n/a	Involved in the study	
☐	☒	Antibodies
☐	☒	Eukaryotic cell lines
☒	☐	Palaeontology and archaeology
☐	☒	Animals and other organisms
☒	☐	Clinical data
☒	☐	Dual use research of concern
☒	☐	Plants

Methods

n/a	Involved in the study	
☒	☐	ChIP-seq
☐	☒	Flow cytometry
☒	☐	MRI-based neuroimaging

Antibodies

Antibodies used	Antibodies used in WB:<br>SARS-CoV-2 Spike RBD Antibody,Rabbit PAb (SinoBiological, cat: 40592-T62);<br>Anti-rabbit IgG-HRP (abcam, cat: AB6721);<br>Influenza A Virus Hemagglutinin / HA Antibody, Rabbit MAb (SinoBiological, cat:86001-RM01).<br><br>Antibodies used in ELISA:<br>Anti-mouse IgG-HRP (abcam, cat: ab6789);<br>Anit-mouse IgA-HRP (Southern Biotech, cat: 1040-05).<br><br>Antibodies used in Live virus neutralization:<br>Rabbit anti-SARS-CoV nucleocapsid protein antibody (Abclonal, cat: A21042);<br>HRP-conjugated anti-rabbit IgG (Proteintech, cat: SA00001-2).<br><br>Intracellular cytokine staining:<br>PerCP-Cy5.5-CD3 (BioLegend, cat: 100328);<br>APC-eF780-CD4 (Invitrogen, cat: 47-0041-82);<br>AF700-CD8 (BioLegend, cat: 100730);<br>FITC-CD44 (Invitrogen, cat: 1-0441-85);<br>APC-IFNγ (BioLegend, cat: 505810);<br>BV421-TNFα (BioLegend, cat: 506328);<br>PE-Cy7-IL-4 (BioLegend, cat: 504118);<br>PE-IL-13 (BioLegend, cat: 159403);
Validation	Validation for each antibody was confirmed by the manufacturer (Invitrogen, BD Pharmigen, SinoBiological, abcam, Proteintech, Abclonal, BioLegend,or Southern Biotech). Documentation is available on their website.

Eukaryotic cell lines

Policy information about cell lines and Sex and Gender in Research

Cell line source(s)	HEK-293, 293T, Huh7, MDCK, and VeroE6 cells were all obtained from ATCC.
Authentication	Cell lines were authenticated by supplier.
Mycoplasma contamination	All cell lines were tested negative for mycoplasma.
Commonly misidentified lines<br>(See ICLAC register)	N/A

Palaeontology and Archaeology

Specimen provenance	N/A
Specimen deposition	N/A
Dating methods	N/A

☐ Tick this box to confirm that the raw and calibrated dates are available in the paper or in Supplementary Information.

Ethics oversight	N/A

Note that full information on the approval of the study protocol must also be provided in the manuscript.

Animals and other research organisms

Policy information about studies involving animals; ARRIVE guidelines recommended for reporting animal research, and Sex and Gender in Research

Laboratory animals	Six- to eight-week-old female C57BL/6J and K18-hACE2 transgenic mice were purchased from Beijing Vital River Laboratory Animal Technology Co., Ltd. (Beijing, China) and GemPharmatech Co., Ltd. (Jiangsu, China), respectively.
Wild animals	N/A
Reporting on sex	Only female mice were used for this study.
Field-collected samples	N/A

Ethics oversight	All studies were conducted under protocols approved by the Institutional Animal Care and Use Committee of Tianjin Medical University and Fudan University.

Note that full information on the approval of the study protocol must also be provided in the manuscript.

Clinical data

Policy information about clinical studies

All manuscripts should comply with the ICMJE guidelines for publication of clinical research and a completed CONSORT checklist must be included with all submissions.

Clinical trial registration	N/A
Study protocol	N/A
Data collection	N/A
Outcomes	N/A

Dual use research of concern

Policy information about dual use research of concern

Hazards

Could the accidental, deliberate or reckless misuse of agents or technologies generated in the work, or the application of information presented in the manuscript, pose a threat to:

No	Yes	
☒	☐	Public health
☒	☐	National security
☒	☐	Crops and/or livestock
☒	☐	Ecosystems
☒	☐	Any other significant area

Experiments of concern

Does the work involve any of these experiments of concern:

No	Yes	
☒	☐	Demonstrate how to render a vaccine ineffective
☒	☐	Confer resistance to therapeutically useful antibiotics or antiviral agents
☒	☐	Enhance the virulence of a pathogen or render a nonpathogen virulent
☒	☐	Increase transmissibility of a pathogen
☒	☐	Alter the host range of a pathogen
☒	☐	Enable evasion of diagnostic/detection modalities
☒	☐	Enable the weaponization of a biological agent or toxin
☒	☐	Any other potentially harmful combination of experiments and agents

Plants

Seed stocks	N/A
Novel plant genotypes	N/A
Authentication	N/A

ChIP-seq

Data deposition

☐ Confirm that both raw and final processed data have been deposited in a public database such as GEO.

☐ Confirm that you have deposited or provided access to graph files (e.g. BED files) for the called peaks.

Data access links<br>*May remain private before publication.*	N/A
Files in database submission	N/A
Genome browser session (e.g. UCSC)	N/A

Methodology

Replicates	N/A
Sequencing depth	N/A
Antibodies	N/A
Peak calling parameters	N/A
Data quality	N/A
Software	N/A

Flow Cytometry

Plots

Confirm that:

☒ The axis labels state the marker and fluorochrome used (e.g. CD4-FITC).

☒ The axis scales are clearly visible. Include numbers along axes only for bottom left plot of group (a 'group' is an analysis of identical markers).

☒ All plots are contour plots with outliers or pseudocolor plots.

☒ A numerical value for number of cells or percentage (with statistics) is provided.

Methodology

Sample preparation	Splenocytes and leukocytes of the lungs were isolated 4 weeks after last immunization. Mouse lung was dissected, minced, and incubated in RPMI containing DNase I and collagenase. Leukocytes were isolated from the single cell suspension by Percoll density gradient centrifugation. Splenocytes were prepared by grinding between two layers of nylon filters. Red blood cells of spleen and lung were lysed with ACK lysis buffer. After filtering by a 40 µm cell strainer, isolated cells were placed in 96-well plate and stimulated with 2 µg/mL of the RBD, TCEs, or HA pool in the presence of GolgiPlug for 12 h. Upon stimulation, cells were blocked with Fc block (BD Biosciences, Franklin Lakes, NJ, USA) and simultaneously stained with LIVE/DEAD Fixable dye (Thermo Fisher Scientific, Waltham, MA, USA) at 4 °C for 30 min. Cells were then stained with four surface markers containing PerCP/Cyanine5.5-CD3ε, APC-Cy7-CD4, Alexa Fluor 700-CD8a, and FITC-CD44 for 30 min at 4 °C, followed by fixation and permeabilization using fixation/permeabilization solution (BD Biosciences, San Jose, CA, United States) following the manufacturer's instruction. Fixed/permeabilized cells were incubated with a cocktail of fluorescently labeled cytokine antibodies containing APC-IFN-γ, Brilliant Violet 421-TNF-α, PE-IL-13, and PE-Cy7-IL-4 for 30 min at 4 °C. Finally, samples were fixed with 2% PFA for 30 min before analysis.
Instrument	LSRForterssa (BD Biosciences)
Software	Data was analyzed on FlowJo v10 software.
Cell population abundance	Cell population abundance is shown in Supplementary Fig. 2.
Gating strategy	After gating for live lymphocytes in forward and side scatter, cell aggregates/doublets were ignored. T cells (CD3+) were stratified into CD4 T cells (CD4+) and CD8 T cells (CD8+), and their subsets were analyzed for the expression levels of IFNγ, TNFα, IL-4, and IL-13.

☒ Tick this box to confirm that a figure exemplifying the gating strategy is provided in the Supplementary Information.

Magnetic resonance imaging

Experimental design

Design type	N/A
Design specifications	N/A
Behavioral performance measures	N/A

Acquisition

Imaging type(s)	N/A
Field strength	N/A
Sequence & imaging parameters	N/A
Area of acquisition	N/A

Diffusion MRI ☐ Used ☒ Not used

Preprocessing

Preprocessing software	N/A
Normalization	N/A
Normalization template	N/A
Noise and artifact removal	N/A
Volume censoring	N/A

Statistical modeling & inference

Model type and settings	N/A
Effect(s) tested	N/A

Specify type of analysis: ☐ Whole brain ☐ ROI-based ☐ Both

Statistic type for inference (See Eklund et al. 2016)	N/A
Correction	N/A

Models & analysis

n/a	Involved in the study
☒	☐ Functional and/or effective connectivity
☒	☐ Graph analysis
☒	☐ Multivariate modeling or predictive analysis

Functional and/or effective connectivity	*Report the measures of dependence used and the model details (e.g. Pearson correlation, partial correlation, mutual information).*
Graph analysis	*Report the dependent variable and connectivity measure, specifying weighted graph or binarized graph, subject- or group-level, and the global and/or node summaries used (e.g. clustering coefficient, efficiency, etc.).*
Multivariate modeling and predictive analysis	*Specify independent variables, features extraction and dimension reduction, model, training and evaluation metrics.*
